# Walking like a robot: do the ground reaction forces still intersect near one point when humans imitate a humanoid robot?

**DOI:** 10.1098/rsos.221473

**Published:** 2023-05-31

**Authors:** Johanna Vielemeyer, Nora-Sophie Staufenberg, Lucas Schreff, Daniel Rixen, Roy Müller

**Affiliations:** ^1^ Institute of Sport Sciences, Friedrich-Schiller-University Jena, 07737 Jena, Germany; ^2^ GaitLab, Klinikum Bayreuth GmbH, 95445 Bayreuth, Germany; ^3^ Munich Institute of Robotics and Machine Intelligence, Technical University Munich, 85748 Garching, Germany; ^4^ Bayreuth Center of Sport Science, University of Bayreuth, 95447 Bayreuth, Germany

**Keywords:** bipedal gait, humanoid robot, virtual pivot point

## Abstract

Bipedal walking while keeping the upper body upright is a complex task. One strategy to cope with this task is to direct the ground reaction forces toward a point above the centre of mass of the whole body, called virtual pivot point (VPP). This behaviour could be observed in various experimental studies for human and animal walking, but not for the humanoid robot LOLA. The question arose whether humans still show a VPP when walking like LOLA. For this purpose, ten participants imitated LOLA in speed, posture, and mass distribution (LOLA-like walking). It could be found that humans do not differ from LOLA in spatio-temporal parameters for the LOLA-like walking, in contrast to upright walking with preferred speed. Eight of the participants show a VPP in all conditions (*R*^2^ > 0.90 ± 0.09), while two participants had no VPP for LOLA-like walking (*R*^2^ < 0.52). In the latter case, the horizontal ground reaction forces are not balanced around zero in the single support phase, which is presumably the key variable for the absence of the VPP.

## 1. Introduction

Walking is commonplace in humans, but however not trivial, as the heavy trunk must be balanced. Various balancing strategies are possible, keeping the trunk close to vertical. These strategies can be described in a simplified way with templates [1]. One such template for human walking is the spring-loaded inverted pendulum model with a trunk (TSLIP). This is a lower body model (e.g. [2–4]) extended by a trunk as rigid body (e.g. [5–7]). There exist several connections between lower and upper body for the TSLIP model, e.g. adding compliant hips [8,9] or adjusting the hip torques such that the ground reaction forces (GRFs) intersect at a point, the virtual pivot point (VPP), above the centre of mass (CoM) during one stride [5].

Analogous to the VPP model, an intersection point of the GRFs above the CoM could also be found in various experimental studies for human walking [5,10–14], and even for some animals such as dogs [5,15], macaques [16] and quails [17]. In those experiments, the GRFs point to the VPP with a small spread. In simulations of running humans, humanoid robots and birds, there were stable solutions for the VPP position both below and above the CoM [18–21]. However, the question arises whether the VPP is a target driving the gait strategy or merely a consequence of the complex dynamics and control during gait.

The intersection point is often reported to appear within a large range of heights. Already Maus *et al.* [5] has reported a VPP position of 5–70 cm above the CoM. Additionally, a VPP could also be observed in walking with different trunk inclinations [11] and in walking over visible and camouflaged curbs [12], although the VPP height varied between and within participants. There are control mechanisms in simulation where the VPP emerges purely from mechanics and leg force feedback [8,9]. These observations lead to the assumption that the VPP is not a target variable but emerges as a side product and the position depends on the type of gait.

However, although a VPP has been observed in all known human VPP studies, there are also systems like the humanoid robot LOLA that walk without a VPP. LOLA is stabilized by a real-time controller which uses hybrid force and position control of CoM and foot trajectories (for technical details, see [22–26]). The robot has no VPP, neither as target quantity nor as an emergent dynamical consequence [27]. LOLA’s gait pattern differs from that of humans in several parameters: its posture is more crouched than a human’s posture to avoid singularities in joint angles and with 0.5 m s^−1^ it walks significantly slower than the preferred human walking speed. Additionally, LOLA has a different mass distribution with its legs being relatively heavier than those of humans. At least the first two changes in gait are also of clinical interest, as various diseases (e.g. cerebral palsy [28]) and the behaviour of the elderly (e.g. [29]) may be associated with these traits.

The question is whether and how these changed parameters affect the absence of VPP, leading to the following research question: When humans imitate LOLA’s gait in speed, posture, and weight distribution all at the same time (LOLA-like walking), do gait parameters more closely resemble those of LOLA and can a VPP still be observed? Based on former studies, it is assumed here that the VPP occurs as a mechanical consequence [11,12,30], and thus can be considered independently from the most likely different control strategies of humans and robot.

We hypothesize that the LOLA-like walking of humans would affect both the position of the VPP and the spread around that point. Here, the spread is likely to increase, possibly even so far that a VPP can no longer be observed. If there is no VPP in LOLA-like walking anymore, we may get a deeper understanding of the parameters that are responsible for the VPP. If there is still a VPP, differences between humans and robot in VPP-relevant parameters can be instructive to better understand the mechanics of bipedal walking.

## 2. Material and methods

### 2.1. Participants

Eleven participants took part in this experiment. Data from a single participant have been discarded due to incomplete kinematic data; therefore trials from ten volunteers (two female, eight male; mean ± s.d., average age: 30.7±10.5 years, age range: 23–57 years, mass: 74.4 ± 15.3 kg, height: 1.78 ± 0.07 m) were considered in the analysis. All participants were physically active and had no known limitations which could have affected their performance in the study. Prior to participation, each volunteer signed an informed consent form. The experiment was approved by the ethics committee of the University of Jena (3532-08/12), and conducted in accordance with the Declaration of Helsinki.

### 2.2. Measurements

The participants were asked to walk along a 5 m walkway with gait modifications concerning walking speed, posture and weight distribution. Two speeds were conducted: a preferred walking speed (approx. 1.3 m s^−1^) and a slow walking speed comparable to the typical walking speed of the robot LOLA (approx. 0.5 m s^−1^). The speed was controlled with a light barrier system (Witty, Microgate, Bolzano, Italy). Besides the upright, human-like posture, a crouched, robot-like posture was investigated, which was demonstrated by the examiner. The participants were instructed to walk with bent leg joints and arms in a rather stiff gait. This posture was controlled by the examiner through observation and feedback (especially by the reference to bent knees). As illustration for the gait of LOLA, a video of the walking robot was shown to the participants before investigation.

**Figure 1.**
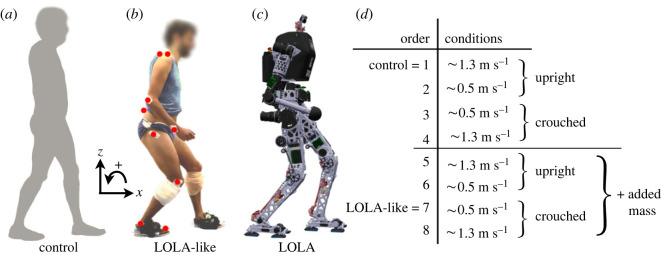
Experimental performance of one participant and robot LOLA. (*a*) Upright walking with preferred speed without added mass (control) and (*b*) crouched slow walking with added mass on shanks and feet (LOLA-like). Red circles indicate the positions of the markers on one body side. (*c*) Robot LOLA during walking. (*d*) Overview of the measurement order.

Firstly, the participants had to walk with the preferred speed and an upright posture (control condition; figure 1*a*). This was followed by walking slowly, first with an upright posture and second with a crouched posture. Thereafter, the crouched posture was performed at the preferred walking speed. Then these four settings were repeated with weights added on the participant’s legs. Figure 1*d* shows the order of performance. Weights were attached to shanks and feet (figure 1*b*) and selected for each participant to match robot LOLA’s weight distribution. For details concerning the calculation of added weight, see table S1 in the electronic supplementary material. Several practice trials took place before each setting until the participant could adequately perform the movement task. Figure 1*b* illustrates slow crouched walking with added mass (LOLA-like condition); figure 1*c* shows robot LOLA during walking.

Four force plates (Kistler, Winterthur, Switzerland) were built into the walkway (figure S1; electronic supplementary material). The first two force plates (Type 9260AA6) were aligned along their long sides and rotated clockwise by $41^{\circ }$ around the vertical axis, so that the robot LOLA could place successive contacts on individual force plates. Force plates 3 (Type 9260AA6) and 4 (Type 9286BA) were aligned along their short sides without rotation, i.e. straight behind each other. The GRFs of all force plates were sampled at 250 Hz. The participants were instructed to use force plates 1 and 2 for the slow speed conditions and force plates 3 and 4 for the preferred speed conditions. For each condition, one contact was evaluated.

All trials were recorded with 10 cameras (250 Hz) by a 3D infrared system (Vicon, Oxford, UK). The measurement systems (force plates and cameras) were synchronized using the trigger of the camera system. Depending on performance, participants completed 5–10 trials for each condition. A trial was only analysed when the participant hit each relevant force plate with only one foot without visual targeting of the force plates and without preparatory adjustments of the step length. It was also necessary to maintain the correct speed and correctly realize the above-mentioned instructions without losing any reflective joint marker of the infrared system. The spherical markers (14 mm in diameter) were placed on the tip of the fifth toe, lateral malleolus, epicondylus lateralis femoris, trochanter major, acromion, epicondylus lateralis humeri and ulnar styloid processus on both sides of the body as well as on L5 and C7 process spinosus (figure 1*a*).

For the robot LOLA, kinematic data were collected from the angle sensors of the joints. Kinetic data were measured with the first two force plates and redundantly with internal sensors. Only the first contact was taken into account in the evaluation. Eight runs were performed.

### 2.3. Data processing and statistical analysis

Human raw data were filtered with a fourth-order bidirectional low-pass Butterworth filter. Human kinetic data were filtered at a cutoff frequency of 30 Hz and their kinematic data at a cutoff frequency of 50 Hz. GRFs of humans and robot were normalized to individual body weight (BW). The instances of touch down (TD) and take-off (TO) of the first and second contacts were calculated as the events when the GRFs exceeded or fell below the threshold of 0.05 BW. In the absence of additional force plates, the TO of the step before the first contact and the TD of the step after the second contact were calculated using a characteristic apex in the velocity profile of the malleolus lateralis for the human experiments [31]. For the robot, the internal force sensors were used here. The human CoM was determined using a body segment parameters method according to Plagenhoef *et al*. [32].

To calculate the VPP position, GRF vectors starting at the centre of pressure (CoP) were used for every instant of measurement. They were regarded in a CoM-centred coordinate frame, where the vertical axis is parallel to gravity. The position of the VPP with respect to the CoM is defined as the point where the sum of the squared perpendicular distances to the GRFs from TO to the following TD is minimal. The theoretical forces are the linear connections between the CoP and the computed VPP. For estimation of the amount of agreement between theoretical forces and experimentally measured GRFs, the angle of the GRFs *θ*_Exp_ and of the theoretical forces *θ*_VPP_ relative to the ground was considered for each trial (*N*_Trial_) and measurement time (*N*_Time_). The mean experimental angle ${\bar{\theta }}_{\mathrm{Exp}}$ is the grand mean over all trials and measurement times. Then, the coefficient of determination *R*^2^ was calculated as follows, adapted from Herr & Popovic [33]:

2.1 $$R^{2}=1-\frac{\sum_{i=1}^{N_{\mathrm{Trial}}}\sum_{\;j=1}^{N_{\mathrm{Time}}}{\left(\theta_{\mathrm{Exp}}^{ij}-\theta_{\mathrm{VPP}}^{ij}\right)}^{2}}{\sum_{i=1}^{N_{\mathrm{Trial}}}\sum_{\;j=1}^{N_{\mathrm{Time}}}{\left(\theta_{\mathrm{Exp}}^{ij}-{\bar{\theta }}_{\mathrm{Exp}}\right)}^{2}},$$

with at least one pair of *i*, *j*, so that $\theta_{\mathrm{Exp}}^{ij}\neq {\bar{\theta }}_{\mathrm{Exp}}$. The VPP as well as the *R*^2^ were calculated for the exact single support phase, as described in Vielemeyer *et al.* [13].

By definition, the values of *R*^2^ can vary between −∞ and 1. Note that an *R*^2^ value of 1 indicates a perfect fit between model and experiment and an *R*^2^ value of 0 or even negative values mean that the estimation of the model is equal to or worse than using the mean experimental value as an estimate [33]. Based on the rating of Herr & Popovic [33], the VPP was defined as a point if *R*^2^ was greater than 0.6, separately for each condition. The VPP position was only calculated if it was classified as a point. Here, the anterior–posterior (*x*) direction and the vertical (*z*) direction were considered. The (centroidal) angular momentum of the whole body was calculated as described in Vielemeyer *et al.* [12].

To compare spatio-temporal gait parameters (table 1) and VPP variables (table 2) between conditions, repeated measures ANOVA (*p* < 0.05) regarding the factors ‘speed’ (preferred and slow), ‘posture’ (upright and crouched) and ‘mass’ (without and with added mass) were used. To examine whether the variables differ between humans and LOLA, one-sample *t*-tests between humans and LOLA were conducted separately for each condition. To analyse whether the VPP was above, below or at the CoM, and anterior or posterior to it, *t*-tests compared to zero were performed separately for each condition.

**Table 1.** Statistical analysis of spatio-temporal gait parameters. Human data are mean ± s.d. between participants. Significant differences between humans and LOLA are underlined, significant *p*-values are in bold. Only significant interactions are shown. The set-ups are preferred speed with/without added mass and slow with/without added mass. The speed is calculated as mean value of one contact. Additionally, contact time, step length, double support phase (DSP) time and single support phase (SSP) time are shown. ‘rel’ is the relative duration of the corresponding phase with respect to the contact time.

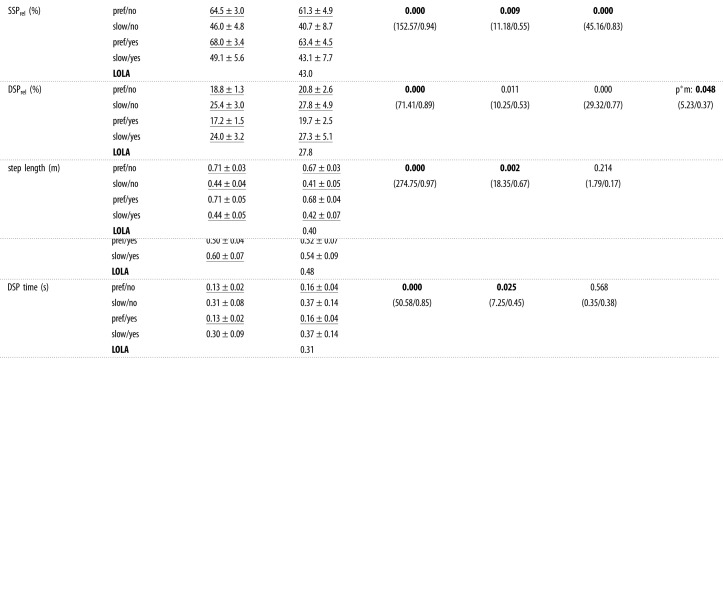

**Table 2.** Statistical analysis of virtual pivot point (VPP) variables. Human data are mean ± s.d. between participants. Only significant interactions are shown. The set-ups are preferred speed with/without added mass and slow speed with/without added mass. The horizontal (*x*) and vertical (*z*) position of the VPP are calculated for *R*^2^ > 0.6.

		**posture**		***p*-value (*F*-value/*η*^2^)**			
	**speed/mass**	**upright**	**crouched**	**speed (s)**	**posture (p)**	**mass (m)**	**interaction**
*R*^2^	pref/no	0.99 ± 0.00	0.95 ± 0.05	0.246	0.201	0.364	
	slow/no	0.98 ± 0.01	0.21 ± 2.19	(1.54/0.15)	(1.907/0.17)	(0.91/0.09)	
	pref/yes	0.99 ± 0.01	0.97 ± 0.02				
	slow/yes	0.98 ± 0.03	0.82 ± 0.19				
	**LOLA**		−0.86				
VPPx (cm)	pref/no	−3.2 ± 0.9	−5.8 ± 2.2	0.098	0.057	**0.008**	s*p: **0.007**
	slow/no	−3.2 ± 0.9	−4.1 ± 1.9	(3.66/0.34)	(5.17/0.43)	(13.25/0.65)	(14.02/0.67)
	pref/yes	−2.7 ± 1.9	−4.4 ± 1.8				
	slow/yes	−3.5 ± 1.2	−3.6 ± 1.9				
	**LOLA**		—				
VPPz (cm)	pref/no	27.7 ± 7.6	64.9 ± 23.9	0.000	0.006	0.000	s*m: **0.002**
	slow/no	48.7 ± 15.7	72.5 ± 21.6	(43.98/0.86)	(15.56/0.69)	(126.43/0.95)	(22.47/0.76)
	pref/yes	41.0 ± 9.7	81.5 ± 26.5				p*m: **0.043**
	slow/yes	78.7 ± 26.0	140.0 ± 47.7				(6.12/0.47)
	**LOLA**		—				

## 3. Results

**Figure 2.**
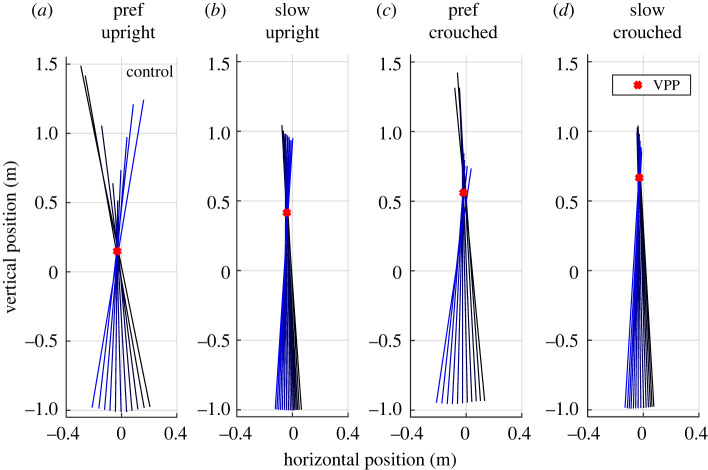
Exemplary plot of the virtual pivot point (VPP). VPP of a representative participant for experimental set-ups without added mass is shown. (*a*) Preferred speed, upright posture, (*b*) slow speed, upright posture, (*c*) preferred speed, crouched posture, (*d*) slow speed, crouched posture. Coloured lines show the ground reaction forces (GRFs) scaled with factor two at different measurement times originating at the centre of pressure in a coordinate system centred on the centre of mass. The illustration of the GRFs starts at touch down (black) and ends at take-off (blue). Red crosses indicate the calculated VPP.

In all investigated spatio-temporal gait parameters (step length, speed, contact time, absolute and relative duration of single support phase, and double support phase) significant differences between human participants and the robot LOLA could be observed for the control condition (upright walking at a preferred speed and without added mass), but not for the LOLA-like condition (crouched slow walking with added mass), as shown in table 1. Furthermore, a mean *R*^2^ value of >0.90 ± 0.09 for eight of ten participants indicates that, contrary to the robot LOLA, these participants have a VPP in all conditions, especially for LOLA-like walking (table 2). The VPP plot does not change strongly when imitating LOLA’s gait, as figure 2 illustrates for one representative participant. The illustration with added mass looks similar and can be found in the electronic supplementary material (figure S2). Since LOLA has a negative *R*^2^ value (table 2), the VPP cannot be denoted as a point, and therefore the VPP position was not calculated.

### 3.1. Virtual pivot point parameters

**Figure 3.**
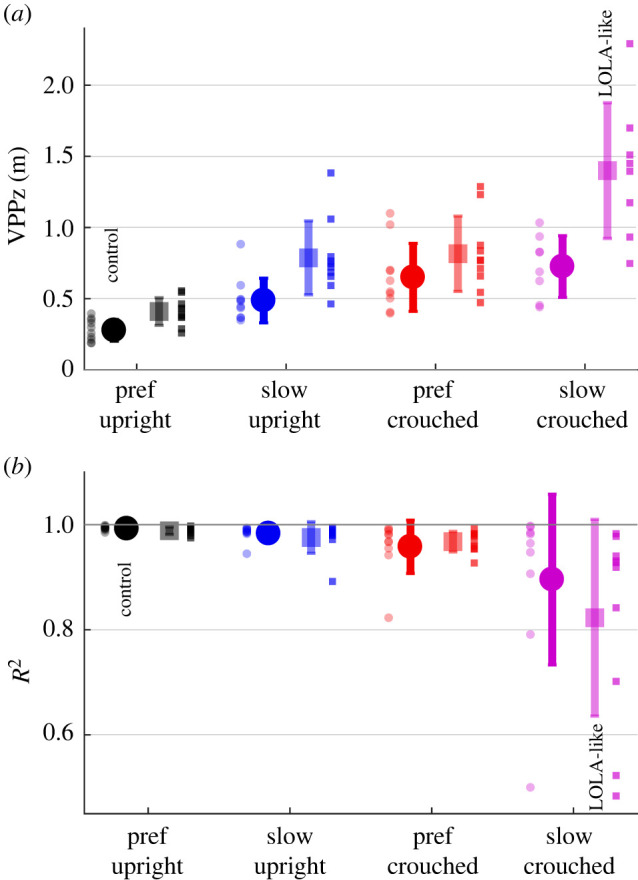
Mean ± s.d. of the virtual pivot point (VPP) variables between participants (*n* = 10) for each experimental set-up (preferred or slow speed, upright or crouched posture, with or without added mass). (*a*) Vertical (*z*) VPP position. Each small dot is the mean over all trials of one set-up for one participant. Values for *R*^2^ < 0.6 were excluded, since below the VPP is considered not to exist (i.e. *n* = 8 for slow crouched with and without added mass). (*b*) *R*^2^ describes the spread around the VPP. Each small dot represents one participant. Note that *R*^2^ values smaller than zero are disregarded for clearness in the plot (i.e. *n* = 9 for slow crouched with added mass), in table 2 all values are shown. Non-transparent circle: without added mass; transparent square: with added mass.

The mean vertical VPP position of humans ranges from 27.7 cm in the control condition to 140.0 cm in the LOLA-like condition, as illustrated in figure 3 and table 2. It is always located significantly above the CoM. All changes in speed, posture, and mass shift the VPP upwards. There are interactions between speed and mass and between posture and mass (table 2). The standard deviation increases analogously. The mean horizontal VPP position of humans was significantly posterior to the CoM in all conditions ranging from −2.7 to −5.8 cm. No significant difference was found in *R*^2^ values between conditions.

**Figure 4.**
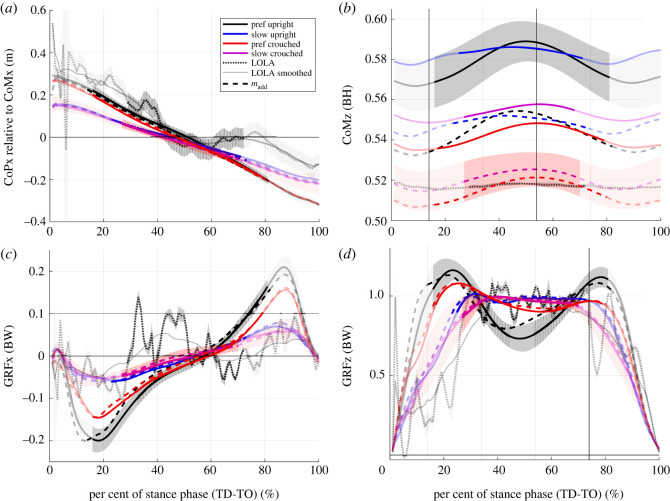
Evolution over time of variables included in the calculation of the virtual pivot point (VPP) from touch down (TD) to take-off (TO). All experimental set-ups (preferred speed upright (black), slow speed upright (blue), preferred speed crouched (red), slow speed crouched (purple), with added mass (dashed line)), and the robot LOLA (grey; dotted line: measured data; solid line: smoothed data) are shown. Values are mean of all trials and subsequent mean of all participants (*n* = 10). For control condition (preferred speed, upright, without added mass) and LOLA-like walking (slow crouched walking with added mass) mean ± s.d. is shown and for the robot LOLA, mean ± s.d. of all trials is illustrated. The non-transparent trajectory represents the single support phase, which is included in the calculations of the VPP. (*a*) Horizontal (*x*), centre of mass (CoM)-related centre of pressure (CoP) position. (*b*) Vertical (*z*), CoP-related CoM position proportional to body height (BH). (*c*) Horizontal ground reaction forces (GRFs) and (*d*) vertical GRFs proportional to body weight (BW). Note that the trajectories of the robot LOLA are noisy due to its control pattern.

The evolution in time of the CoP relative to the horizontal CoM position (CoP_CoM_) differs between humans and robot in all conditions (figure 4*a*); the profiles of the human participants are smoother than the CoP_CoM_ profile of LOLA. To evaluate the difference between the profiles of humans and robot, the difference between the time integrals from TD to TO was calculated. For this integral, no significant differences could be observed between all conditions (table S2; electronic supplementary material). Since the CoM height and peak-to-peak amplitude (figure 4*b*) get smaller in LOLA-like walking compared with the control condition, the profile of the CoM fits better between humans and LOLA in LOLA-like walking than in the control condition. The profiles are consistent within the standard deviation. Here, the slower speed minimizes the peak-to-peak amplitude of CoMz for humans, while posture and added masses minimize its height. Nevertheless, the human CoMz peak-to-peak amplitude is higher than that of LOLA in all conditions. The GRF profiles of humans (figure 4*c*,*d*) are also smoother than that of LOLA, and there is no correspondence of the profiles. Note that this can be observed for single trials as well as for the mean. Nevertheless, all changes in speed, posture, and mass bring the human GRF profiles closer to LOLA’s (smoothed) profile, figure 4, which is also reflected in the integrals displayed in table S2, electronic supplementary material.

All differences between the profiles are significantly greater than zero (*p* < 0.015), i.e. human profiles differ from the profiles of LOLA in all conditions. The differences between the integrals of the input variables of the VPP (CoP, CoM, and GRFs) of humans and robot are smallest for LOLA-like walking or no significant difference can be detected from the smallest value. This means that in LOLA-like walking, one observes the best match of all conditions regarding the input variables of the VPP between LOLA and humans. Electronic supplementary material, table S2, shows values for the whole contact phase, but the ratios are the same for the single support phase.

### 3.2. Participants 5 and 9

**Figure 5.**
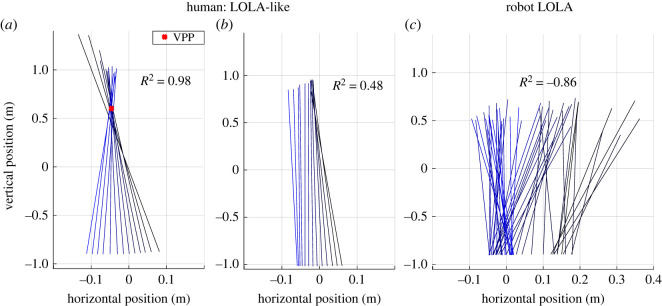
Virtual pivot point (VPP) plot: comparison of robot LOLA and humans. Coloured lines show the ground reaction forces (GRFs) scaled with factor two at different measurement times from touch down (black) to take-off (blue), originating at the centre of pressure in a coordinate system centred on the centre of mass. Red crosses indicate the calculated VPP if *R*^2^ > 0.6. (*a*) One LOLA-like trial (i.e. slow crouched walking with added mass) of participant 3 with *R*^2^ = 0.98, and (*b*) of participant 5 with *R*^2^ = 0.48. (*c*) One trial of robot LOLA. Note that only one *R*^2^ value is calculated for all trials of one condition and therefore the plots of the different trials may vary. Only exemplary plots are shown here, which are representative for all trials.

The *R*^2^ values of two male participants are smaller than 0.6 in some conditions. For the crouched slow walking, participant 5 has *R*^2^ values of about 0.50 and participant 9 has values of −6.00 (without added mass) and 0.52 (with added mass, i.e. LOLA-like condition). That means that no VPP was found here. For all other conditions, these two participants have *R*^2^ values >0.89 and thus a VPP. Figure 5 shows how the VPP plot changes according to *R*^2^ for humans (figure 5*a*,*b*) and for LOLA (figure 5*c*).

**Figure 6.**
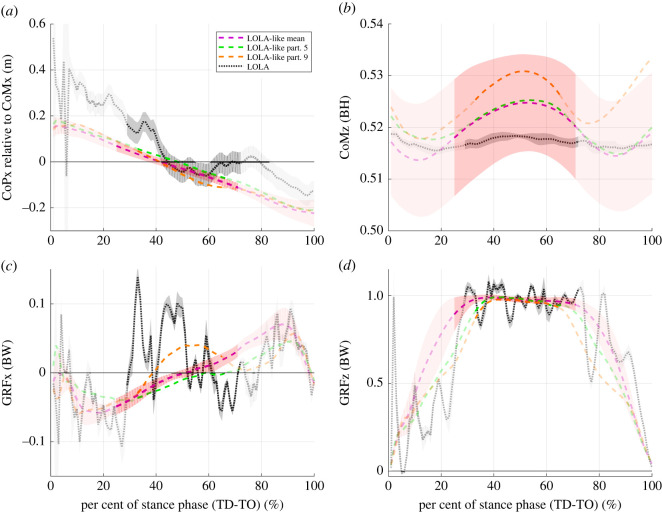
Evolution over time of variables included in the calculation of the virtual pivot point (VPP) for LOLA-like condition (i.e. slow crouched walking with added mass) for outliers from touch down (TD) to take-off (TO). Mean of all trials and subsequent mean ± s.d. of eight participants are illustrated (purple). For the robot LOLA, mean ± s.d. of all trials are shown (grey dotted line). The mean value of all LOLA-like trials is displayed in green for participant 5 and in orange for participant 9. The non-transparent trajectory represents the single support phase, which is included in the calculations of the VPP. (*a*) Horizontal, centre of mass (CoM)-related centre of pressure (CoP) position. (*b*) Vertical (z), CoP-related CoM position proportional to body height (BH). (*c*) Horizontal ground reaction forces (GRFs) and (*d*) vertical GRFs proportional to body weight (BW).

The deviations in CoP_CoM_ and CoMz are within the standard deviation of all participants (figure 6*a*,*b*) and, thus, the influence on the change of *R*^2^ seems to be small. In all conditions and for LOLA, the vertical GRFs are close to one in the single support phase, as shown in figure 6*d*, and thus do not affect the *R*^2^ value noticeably. The main difference between the outliers and the other participants is found in the horizontal GRFs. While for LOLA-like walking participant 5 has exclusively negative horizontal GRFs in the single support phase and participant 9 has predominantly positive values, the mean profile of the other eight participants is more linear and balanced around zero (figure 6*c*). The duration of the single support phase relative to the contact time is smaller for the outliers (participant 5: 0.34, participant 9: 0.31) than for the other participants (mean 0.46 ± 0.06, all values >0.40) for LOLA-like condition (without added mass analogous).

## 4. Discussion

In the hypothesis, it was assumed that the gait changes would affect both the position of the VPP and the spread around it. It could be observed that indeed all changes affect the VPP position. However, no significant difference in spread could be observed between conditions, which can be attributed to the increasing variability between participants with more severe gait changes.

### 4.1. Comparison between humans and robot

**Figure 7.**
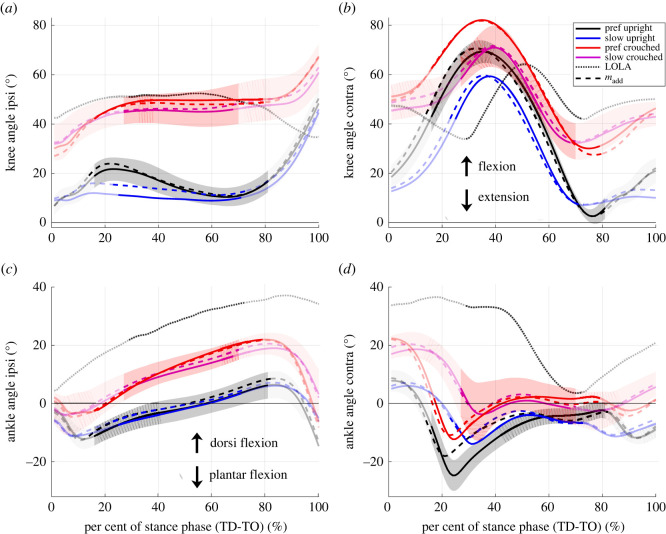
Knee and ankle angles from touch down (TD) to take-off (TO). All experimental set-ups (preferred speed upright (black), slow speed upright (blue), preferred speed crouched (red), slow speed crouched (purple), with added mass (dashed line)) are shown. Values are means of all trials and subsequent mean of all participants (*n* = 10). For control condition (preferred speed, upright, without added mass) and LOLA-like walking (slow crouched walking with added mass) mean ± s.d. is shown and for the robot LOLA, mean ± s.d. of all trials is illustrated (grey dotted line). The non-transparent trajectory represents the single support phase. Ipsi denotes the ipsilateral leg in contact, contra refers to the contralateral leg.

Spatio-temporal gait parameters match between humans and the humanoid robot LOLA for the LOLA-like walking but not for the control condition. This means that the test instructions were suitable to mimic LOLA not only in step length and speed, but also in contact time and duration of single and double support. Furthermore, the knee and ankle angle ranges of the ipsilateral leg fit better between LOLA and humans in the LOLA-like condition than in the control condition, as illustrated in figure 7. There was no fit between humans and robot in the VPP input parameters (CoP_CoM_, CoM, and GRFs), since the profiles differ significantly from zero. However, at least the profiles for CoM and GRFs fit better in LOLA-like walking than in the control condition, and no differences between the conditions for CoP_CoM_ could be observed. The relative duration of single and double support shows similarities between LOLA and humans in LOLA-like condition. However, the profiles of the horizontal GRFs still differ strongly between LOLA and humans (figure 4*c*). Especially in the single support phase, for which the VPP was calculated, the time integral of the horizontal GRFs of LOLA was obviously greater than zero, while in all conditions the human horizontal GRFs are more balanced around zero. These considerable differences in horizontal GRFs are most likely the reason that LOLA has no VPP.

**Figure 8.**
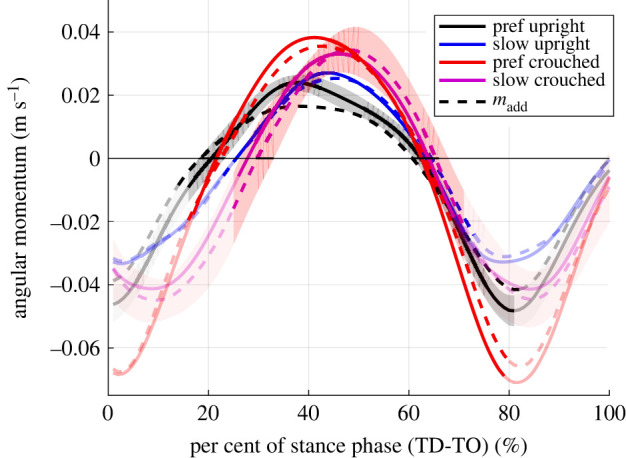
Angular momentum for one contact from touch down (TD) to take-off (TO). All experimental set-ups (preferred speed upright (black), slow speed upright (blue), preferred speed crouched (red), slow speed crouched (purple), with added mass (dashed line)) are illustrated. Values are means of all trials and subsequent mean of all participants (*n* = 10). For control condition (preferred speed, upright, without added mass) and LOLA-like walking (slow crouched walking with added mass) mean ± s.d. is shown. The non-transparent trajectory represents the single support phase. The angular momentum was normalized to participant’s mean centre of mass height and body weight. Negative values indicate clockwise rotation.

Although LOLA has been imitated well in many parameters, there are still crucial differences between the dynamics of LOLA and humans, which remain presumably because of the different control strategies. For the human participants no second peak was found in the vertical GRFs and lower second peaks in the horizontal GRFs for the slow and crouched walking (figure 4). This represents less active ankle plantar flexors for LOLA-like walking than in the control condition [34,35], which is also illustrated in figure 7*c*. However, at slow walking speed, humans make adjustments of the lower limb systems to maintain similar effective foot roll-over geometries as at preferred speed [36]. LOLA, on the other hand, has no roller foot and thus no roll-over geometry, while the low ankle plantar flexion is reflected in the GRFs, similar to the LOLA-like condition in humans (figure 7*c*). Gruben & Boehm [30] found that hip and knee torque control may be adequate for walking upright, but the angular momentum is smaller than with foot roll-over (i.e. ankle torque control). The ankle torque serves for error corrections of the gait [30] and the ankle push-off powers leg swing in human walking [37]. Since LOLA has no swing leg retraction, which could increase the stability [38], there seem to be major differences in the swing leg behaviour between LOLA and normal human walking. This may cause an advantage in stability in humans. Nevertheless, in LOLA-like walking, the angular momentum is not smaller than in the control condition, as illustrated in figure 8. This suggests that the foot roll-over geometry is preserved for LOLA-like walking and thus similar swing leg behaviour can be observed. The results of Browning *et al.* [39], showing that the peak ankle moments in the single support phase do not change when masses are added, support this assumption. Another major difference between humans and robot concerns the anterior–posterior trunk movement. Humans oscillate the trunk near the vertical [12,40,41], while the planned motion for LOLA predetermines a vertical trunk. The peak-to-peak amplitude of the anterior–posterior trunk movement becomes even larger during LOLA-like walking ($3.1\pm 0.9^{\circ }$) than in control condition ($2.5\pm 0.8^{\circ }$), with mass (*p* = 0.024) and posture (*p* = 0.030) having a significant influence. In summary, this means that at least the swing leg retraction, the roller foot and the trunk movement would have to be adapted to LOLA to achieve a more LOLA-like gait in humans.

### 4.2. Virtual pivot point in LOLA-like walking

For eight of 10 participants, a VPP could be observed in all conditions. Here, the particular gait changes have different influences on the VPP input variables, but all shift the VPP significantly upwards. Crouched walking with a preferred speed leads to a larger vertical component and a smaller horizontal component in the GRFs. Additionally, the CoMz shifts downward by approximately 5 cm each for the crouched walking and for the walking with added masses compared with the control condition (figure 4*b*). Both effects increase the VPP height [42]. The slower walking speed causes a smaller ratio of horizontal GRFs in GRF magnitude (figure 4*c*,*d*), which shifts the VPP upward [42]. The VPP shift is in contrast to a previous study in which no effect of speed on VPP height was observed [12]. However, on the one hand, the speed differences were smaller there, and, on the other hand, the gait with 0.5 m s^−1^ might be more different to the preferred speed in this study and the examined speeds of the other study. Additionally, the calculated VPP height for slow walking fits well with the results of Gruben & Boehm [10], where the VPPz was 44 ± 13 cm above the CoM, as estimated in Vielemeyer *et al.* [12].

It is remarkable that for VPPz and *R*^2^ the range between the participants increases when the gait is changed, as illustrated in figure 3 (VPPz: from 20.7 cm in control condition to 160.8 cm in LOLA-like condition; *R*^2^: from 0.01 in control condition to 0.50 in LOLA-like condition). It seems that the greater the deviation from normal upright walking the more undirected the forces. However, no increase in dispersion was observed for the VPP input variables (see standard deviation in figure 4), so it is due to the interaction of the variables. This suggests that upright walking represents an optimum of neuromuscular control that always produces a similar pattern of whole-body angular momentum (and thus VPP). This finding fits to previous studies concerning upright walking [5,10,30,33]. In addition, an increasing dispersion between the participants with increasing perturbation of upright gait can also be observed in other studies, e.g. walking down visible and camouflaged curbs [12] or running down camouflaged drops [20].

Nevertheless, two of ten participants (participants 5 and 9) actually succeeded in walking without VPP in the LOLA-like condition. Here, the time integrals of the horizontal GRFs for the single support phase, for which the VPP was calculated, are noticeable. The integral for participant 5 (participant 9) is exclusively negative (positive) with the lowest (highest) mean value over the trials of all participants. This is also reflected in the horizontal GRFs (figure 6*c*). Therefore, it is possible that this participant falls below (exceeds) a threshold, beyond which VPP behaviour changes. Nevertheless, for the stabilization, the whole contact, i.e. single and double support phase, has an influence. Since VPP and *R*^2^ were only calculated for the single support phase, it cannot be assessed reliably if these two participants have a VPP or not in the LOLA-like condition in the whole stance phase. In a simulation study, stable walking without VPP could be found [43]. Here, the horizontal GRFs are also not balanced around zero (positive integral) in the single support phase. However, steady-state walking without acceleration was investigated, the GRFs are balanced for the whole contact phase. The results of the simulation study support the assumption that the two participants of this study also perform a non-VPP gait for LOLA-like walking and that the horizontal GRFs are the crucial variables for the presence or the absence of the VPP.

The analyses suggest that in LOLA-like walking there is greater variability in the input variables of VPP than in the control condition, because this gait was not practiced as much as normal walking. Specifically, the greater fraction of double support phase, the lower CoM due to the crouched position and the added mass, and the lower dynamic of slow walking increase this margin and make the gait possibly more robust against perturbations [44–48], presumably at the expense of efficient gait. For the robot LOLA, these factors are probably necessary to maintain walking without falling, since it does not have for example a roller foot or swing leg retraction for error corrections. Since a VPP occurred for eight of ten participants for LOLA-like walking, the title question may be answered under reservation in the affirmative.

Now, further studies could follow to analyse the role of the VPP and try to find a gait without VPP. If a gait without VPP were to be found, this could suggest that the VPP is a consequence of the complex dynamics and control, not a target driving the gait strategy. It would then presumably be possible to find the cause of the existence or non-existence of the VPP. From this, it could be concluded what function the VPP has for gait. If the VPP is not relevant for stability, it could, for example, bring energetic advantages. Here, the pilot study of Herr & Popovic [33] can be taken up, where walking with exaggerated leg protraction and retraction movements was examined, similar to a military marching gait. There, fluctuations in GRFs could be observed, whereby unbalanced horizontal GRFs in the single support phase are probably a good option to find a non-VPP gait. In addition, further extreme gait changes, e.g. by disturbing the CoP, are conceivable.

## List of symbols and abbreviations

BW: body weight
BH: body height
CoM: centre of mass of the whole body
CoP: centre of pressure
CoP_CoM_: centre of pressure relative to the horizontal CoM position
DSP: double support phase
GRFs: ground reaction forces
*R* ^2^
: coefficient of determination
SSP: single support phase
TD: touch down
TO: take-off
TSLIP: spring-loaded inverted pendulum model with a trunk
VPP: virtual pivot point

## Data Availability

Kinetic and kinematic data are available from the figshare repository: https://doi.org/10.6084/m9.figshare.20381559 [49]. The data are provided in electronic supplementary material [50].
